# Midline carcinoma with t(15;19) and *BRD4-NUT *fusion oncogene in a 30-year-old female with response to docetaxel and radiotherapy

**DOI:** 10.1186/1471-2407-6-69

**Published:** 2006-03-16

**Authors:** Jens Engleson, Maria Soller, Ioannis Panagopoulos, Anna Dahlén, Michael Dictor, Mats Jerkeman

**Affiliations:** 1 Department of Oncology, Lund University Hospital, SE-221 85, Lund, Sweden; 2 Department of Clinical Genetics, Lund University Hospital, SE-221 85, Lund, Sweden; 3 Department of Pathology, Lund University Hospital, SE-221 85, Lund, Sweden

## Abstract

**Background:**

Poorly differentiated midline carcinoma with a translocation between chromosomes 15 and 19, i.e. t(15;19), has been recognized as a distinct clinical entity for over a decade. This tumor affects young individuals, shows a rapidly fatal clinical course despite intensive therapy. The t(15;19) results in the fusion oncogene *BRD4-NUT*. Information concerning treatment of this rare disorder is scarce.

**Case presentation:**

A 30-year-old woman was admitted with a rapidly progressing tumor in the mediastinum, cervical lymph nodes, vertebral column and the epidural space. Pathological, cytogenetic, FISH and PCR analysis revealed a glycogenated carcinoma rarely expressing cytokeratins and showing t(15;19) and *BRD4-NUT *gene rearrangement. The patient was initially treated with a Ewing sarcoma chemotherapy regimen, but had rapid progression after two cycles. She then received docetaxel and radiotherapy, which resulted in almost complete disappearance of the tumor.

**Conclusion:**

Docetaxel may be considered for initial chemotherapy in young patients presenting with a midline carcinoma with bone marrow involvement and cytogenetic and molecular genetic finding of a t(15;19)/*BRD4-NUT-*rearrangement. We herein describe, in detail, the laboratory methods by which the *BRD4-NUT *-rearrangement can be detected.

## Background

In 1991, midline carcinoma associated with a (15;19) translocation was identified in young individuals as a rare but clinically distinct subgroup of poorly differentiated carcinoma [1,2]. It has recently been shown that the translocation results in a novel fusion gene, *BRD4-NUT *[3]. BRD4 (bromodomain containing 4) is a bromodomain protein with an important role in cell cycle regulation inhibiting cell cycle progression from G1 to S, whereas NUT (nuclear protein in testis) is a protein of unknown function, normally only expressed in testis tissue [3]. In a recent series, 8 cases with *BRD4-NUT *fusion oncogene were reviewed. Typically, the patients were children or young adults, and all but one of the tumors originated from the respiratory tract or thymus. Clinically, the patients have shown an average overall survival of only 28 weeks with rapid disease progression and dissemination in spite of intensive chemo- and radiotherapy [4]. In the present report we describe cyto- and molecular genetic methods for the diagnosis of these rare tumors and detailed information about the clinical pathology findings. We also report a remarkable effect of docetaxel chemotherapy in this patient.

## Case presentation

A 30-year-old woman presented with a history of one month of accelerating back pain, 15% weight loss and night sweats. At admission she had palpable cervical lymph nodes. Chest x-ray showed an enlarged mediastinum. A laboratory screening showed a markedly elevated lactate dehydrogenase (LD) of 32.2 μkat/L. The following day she developed the superior vena cava syndrome (VCS) with swelling of the upper extremities and the face. A CT scan confirmed tumor growth compressing the superior vena cava and bilateral pleural effusion. MRI of the spinal cord showed tumor infiltrates in all vertebrae, with epidural tumor growth extending from Th9 to L3. Fine-needle aspiration of a palpable lymph node revealed uncharacteristic largely dissociated medium-sized polygonal tumor cells with clear to eosinophilic cytoplasm and small paranuclear vacuoles. Flow cytometry of a bone marrow aspirate was negative for all hematopoietic markers including CD34. While awaiting definitive pathological diagnosis, the patient was treated with cyclophosphamide, 200 mg/m^2^/day for two days. Steroids and a caval stent alleviated VCS symptoms.

**Figure 1:**
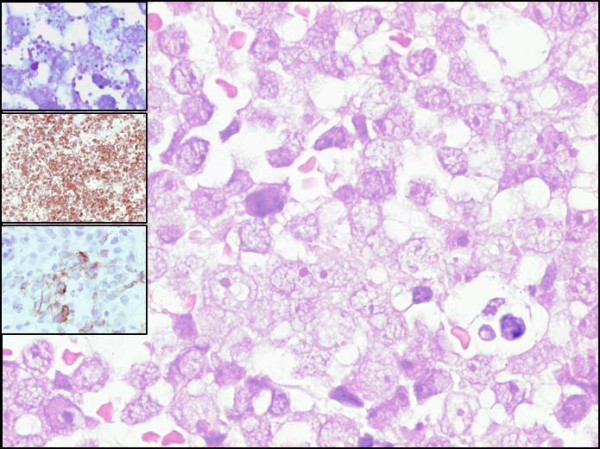
Tumor section (H&E) shows loosely arranged medium-sized cells with finely dispersed chromatin, solitary nucleoli and clear, often vacuolated cytoplasm. The upper left inset depicts PAS-positive cytoplasmic glycogen granules, while the middle inset shows strong epithelial membrane antigen expression in all tumor cells. The lower inset includes the single field which expressed the broad spectrum cytokeratin marker MNF116.

**Figure 2:**
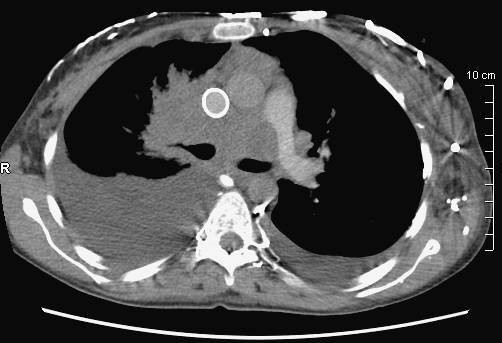
CT-scan of upper mediastinum when the tumor progressed after two cycles of VIDE. Tumor masses almost surrounds the superior vena cava (with the stent) and right upper lobe bronchus. At the autopsy four weeks later, there was only a tiny focus of tumor left in conjunction with the cava stent.

Sections of excised neck tumor displayed PAS-positive cytoplasmic glycogen, which was removed after pre-treatment with diastase. Cell nuclei were large with finely dispersed chromatin and prominent single nucleoli and the cells were loosely arranged in interwoven cords and small dissociated sheets. Initial positive immunostains for CD99 and FLI1 suggested a possibility of metastatic Ewing's sarcoma. Only a minor fraction (<5%) expressed cytokeratins using the MNF116 marker or AE1/AE3, CK5, and CK19 while CK7 and CK20 were negative. EMA was strongly expressed in all tumor cells (figure 1). Immunostains for placental alkaline phosphatase, S-100, CD30, CD34, hematopoietic and common mesenchymal markers were negative. The tumor was regarded as a poorly differentiated glycogen-bearing carcinoma, and the possibility of a thymic origin (clear cell carcinoma) was entertained. However, based on the initial clinical and cytologic impression of possible metastatic Ewing's sarcoma, the patient was treated according to the Euro-E.W.I.N.G protocol, with the VIDE regimen (vincristine 1,5 mg/m^2 ^day 1, ifosfamide 3 g/m^2 ^day 1–3, doxorubicin 20 mg/m^2 ^day 1–3, etoposide 150 mg/m^2 ^day 1–3) q 3 w. Three weeks after the second cycle the patient was admitted due to swelling of the face and neck. CT-scan showed marked progression of mediastinal tumor which compressed vessels and bronchi (Figure 2). Immediate radiation therapy to the mediastinum was begun, 30 Gy in 8 fractions. Weekly docetaxel 30 mg/m^2 ^was given concomitantly, and continued after the radiation therapy, in total two cycles. Face and neck swelling improved, but three weeks after completing radiation therapy and 13 weeks after her first admission to hospital, the patient died suddenly of respiratory failure. Autopsy revealed no tumor remaining in the bone marrow and neck nodes, but a tiny focus remained near the VCS stent. Death was attributed to septicemia.

### Cytogenetic and FISH-analysis

**Figure 3:**
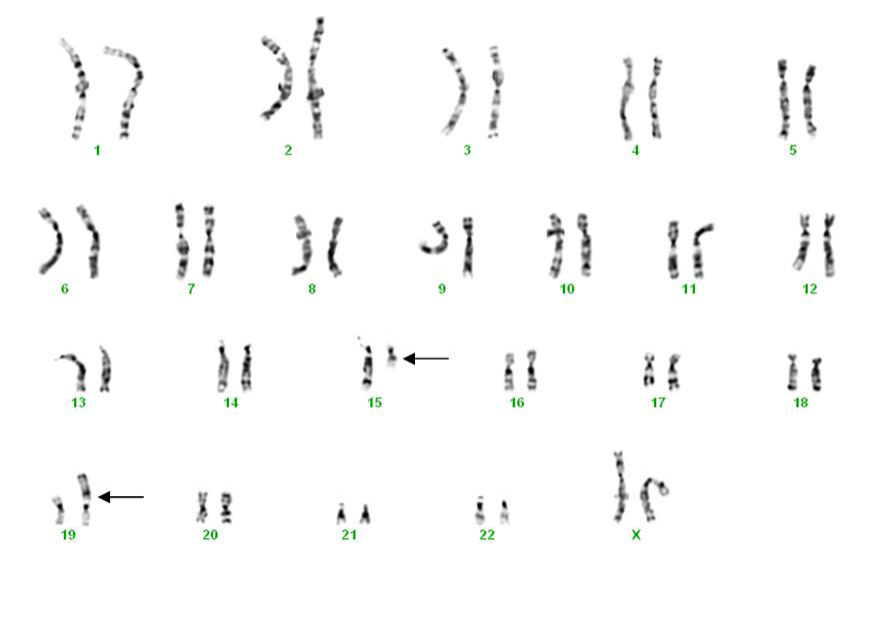
Representative karyogram with the t(15;19)(q14-15;p13). Breakpoints are marked with arrows.

**Figure 4:**
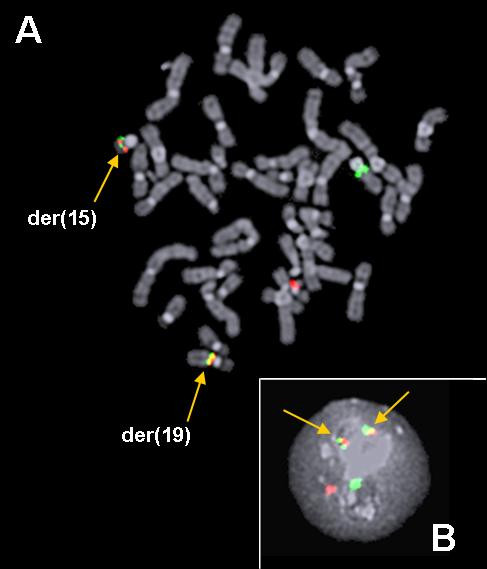
Fluorecense in situ hybridization: a) Metaphase FISH of the t(15;19) showing one green (15q14) and one red (19p13) signal on the normal homologues. The 15q14- and the 19p13-specific BAC probes were both split by the translocation and therefore visualized as a red-green (yellow) fusion signals on der(15) and der(19), respectively. b) Interphase FISH. Yellow arrows indicate fusions.

Cytogenetic analysis of the lymph node as well as bone marrow cells revealed a 46,XX,t(15;19)(q14-15;p13) as the sole clonal change (Figure 3). FISH analyses regarding *EWRS1*-rearrangement (LSI EWSR1 Dual Color Break Apart Rearrangement Probe, Vysis/Abbott, Wiesbaden-Delkenheim, Germany) were negative in both lymph node and bone marrow samples. The t(15;19) was further characterized by metaphase FISH using probes for *BRD4 *and *NUT*. Two bacterial artificial chromosome (BAC) clones, RP11-194H7 and RP11-637P24, covering the *NUT *(15q14) and *BRD4 *(19p13) loci, respectively, were selected from the UCSC Human Genome Browser [5] and provided by the BACPAC Resources Center [6]. The BAC probes were labeled with Cy3-dUTP (Amersham, Buckinghamshire, UK) or fluorescein (FITC)-dUTP (Roche, Mannheim, Germany) and hybridized to metaphase spreads of the excised and cultured tumor tissue. The FISH treatments and analyses were performed as described [7]. Metaphase FISH analysis with the BAC clones RP11-194H7 and RP11-637P24 revealed that both of them were split by the 15;19-translocation. Simultaneous hybridization with RP11-194H7 and RP11-637P24 resulted in one green (15q14) and one red (19p13) signal on the corresponding homologues not involved in the translocation. A red-green (yellow) fusion signal was seen on der(15) and der(19), respectively (Figure 4).

### RT-PCR analysis

RT-PCR was carried out for the detection of the *BRD4-NUT *chimeric transcript. Total RNA was extracted using the Trizol reagent according to the manufacturer's instructions (Invitrogen, Stockholm, Sweden), and 5 μg were reverse-transcribed in a 20 μL reaction volume containing 50 mM Tris-HCl pH 8.3 (at 25°C), 75 mM KCl, 3 mM MgCl_2_, 10 mM DTT, 1 mM of each dNTP, 20 units RNA guard (Amersham Biosciences, Uppsala, Sweden), 10 pmol random hexamers, and 400 units M-MLV Reverse Transcriptase (Invitrogen). The reaction was carried out at 37°C for 60 minutes, heated for 10 minutes at 65°C, and then kept at 4°C until analysis.

**Figure 5:**
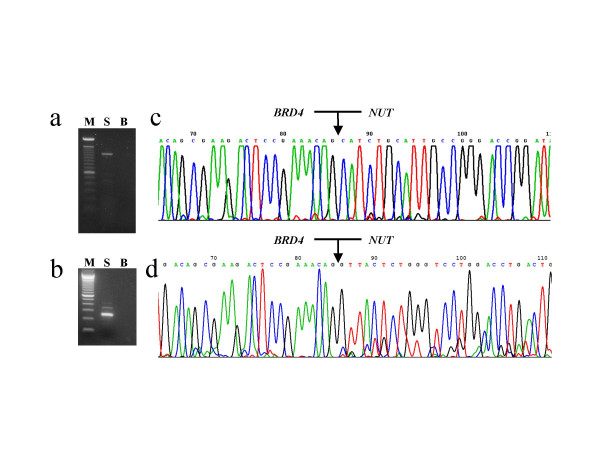
Detection of *BRD4-NUT *chimeric transcript is shown in a-d. Total RNA was reverse transcribed and cDNA was used as a template in (a) PCR amplification with BR2276F and NUT1194R primer combination and (b) PCR amplification with BR2276F and NUT294R primers. M, 100 bp DNA ladder; S, patient with aggressive carcinoma; B, no RNA in the cDNA synthesis. (c) Partial sequence chromatogram showing the in-frame fusion of nt 2380 of *BRD4 *(accession number AF386649) with nt 172 of *NUT *(accession number AF482429). (d) Partial sequence chromatogram showing the out-of-frame fusion of nt 2380 of *BRD4 *with nt 78 of *NUT*. Arrow indicate the junction of the *BRD4 *and *NUT *genes.

PCR amplification was performed using 1 μl of the cDNA as template in 50 μL reaction volume containing 20 mM Tris-HCl (pH 8.4), 50 mM KCl, 1.25 mM MgCl_2_, 0.2 mM of each dNTP, 1 unit Platinum *Taq *polymerase (Invitrogen) and 0.5 μM of each of the primers BR2276F: AAGTTGATGTGATTGCCGGCTCCTC and NUT1194R: GAGGTCTCTGGGCTTTACGCTGACG [3] or BR2276F and NUT294R: CTGAAGGCATGATGGGCTGTGG. The PCR was run on a PCT-200 DNA Engine (MJ Research, Waltham, MA). The cycling profile for the primer set BR2276F/NUT1194R included an initial denaturation at 94°C for 3 min, followed by 30 cycles of 1 min at 94°C, 1 min at 60°C, and 1 min at 72°C, and a final extension for 5 min at 72°C. The cycling profile for the primer set BR2276F/NUT294R was similar except that post-denaturation annealing-extension profiles were reduced to 30 sec. Fifteen microliters of the PCR products were electrophoresed in 1.2% agarose gels, stained with ethidium bromide, and photographed. The amplified cDNA fragments were purified using the Qiagen gel extraction kit (Qiagen, Hilden, Germany), and directly sequenced using the dideoxy procedure with an ABI Prism BigDye terminator v1.1 cycle sequencing kit (PE Applied Biosystems, Foster City, CA). RT-PCR with the the primer set BR2276F/NUT1194R amplified a 1158 bp cDNA fragment (Figure 5a). PCR with the primer set BR2276F/NUT294R generated a strong 255 bp fragment and two weakly amplified bands of 350 and 450 bp (Figure 5b). Direct sequencing of the 1158 and 255 bp fragments showed that nt 2380 of *BRD4 *(accession number AF386649) (figure 5c) was in-frame fused with nt 172 of *NUT *(accession number AF482429). This fusion was identical to that previously reported [3]. Sequence analysis of the 350 bp fragment showed an out-of-frame fusion of nt 2380 of *BRD4 *with nt 78 of *NUT *(Figure 5d).

## Conclusion

The few previously described cases of midline carcinoma were all aggressive tumors with a rapidly fatal clinical course, occurring in young patients. The present case confirms the clinical picture. This rare entity should be considered in the differential diagnosis in young patients with aggressively growing mediastinal tumors, and it may not be initially recognized as a carcinoma, especially if cytokeratin staining is spotty. The diagnosis of such midline tumors requires chromosomal or molecular analysis. The FISH analysis described in this paper may serve as a diagnostic tool in this regard. However, as variant *NUT-*rearrangements have been described [4], FISH techniques that detect both *BRD4-NUT *fusions and variant *NUT*-rearrangements should also be considered. If no fresh tissue is available, interphase FISH analysis on formalin-fixed tissue is a possibility (Figure 4).

There is scarce information concerning treatment in this disorder. Our patient was initially treated with VIDE, based on a clinical and morphological similarity to metastatic Ewing's sarcoma. The patient was improved initially on this regimen, but rapidly worsened after two cycles. The regimen was then altered to weekly docetaxel and there was a remarkable tumor regression after only two weekly courses of docetaxel, the first course given concomitantly with radiation therapy to the mediastinum. The effect was evident both within and outside of the irradiated volume. It is important that new cases with this rare and lethal tumor are reported. Possibly, the BRD4-NUT fusion protein may provide a rational therapeutic target that may improve the prognosis in the future. At present, an initial attempt with docetaxel chemotherapy may be justified.

## Competing interests

The author(s) declare that they have no competing interests.

## Authors' contributions

JE and MJ were involved in the clinical care of the patient, and drafted the manuscript. MS, IP and AD carried out the molecular genetic studies. MD performed the histopathological review and reviewed the immunohistochemical stainings. All authors read and approved the final manuscript

## Pre-publication history

The pre-publication history for this paper can be accessed here:
